# Supplementary figures and images for: Sustaining the T-cell activity in xenografted psoriasis skin
Source: PLoS One. 2023 Jan 17;18(1):e0278390. doi: 10.1371/journal.pone.0278390 (PMC9844869; doi:10.1371/journal.pone.0278390)

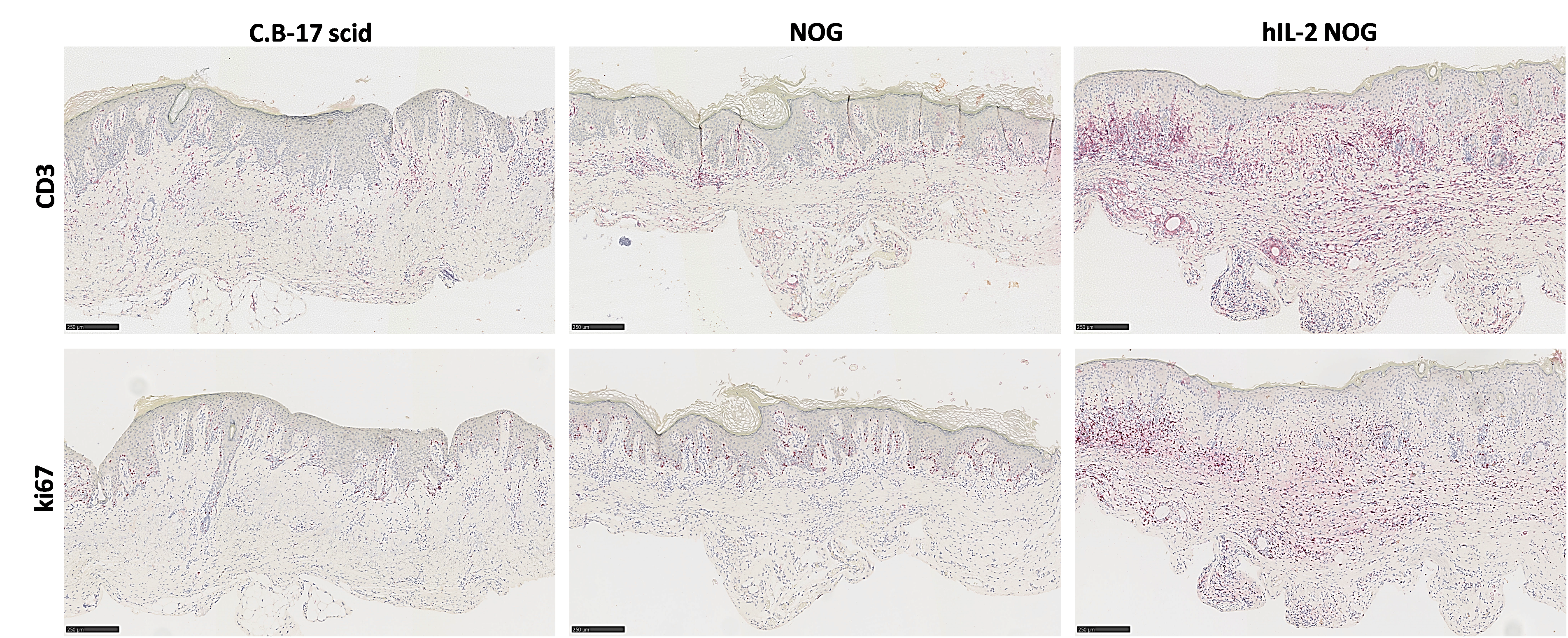

Supplement: S1 Fig — Immunohistochemical stainings with anti-human CD3 and anti-human ki67. Representative slides are shown. The bar equals to 250 μm and slides are shown in a 10X magnification. Keratomes from six psoriasis vulgaris patients were included in the study. The number of mice in each group were C.B-17 scid n = 20; NOG n = 20; hIL2-NOG n = 11. (TIF) [file pone.0278390.s001.tif]

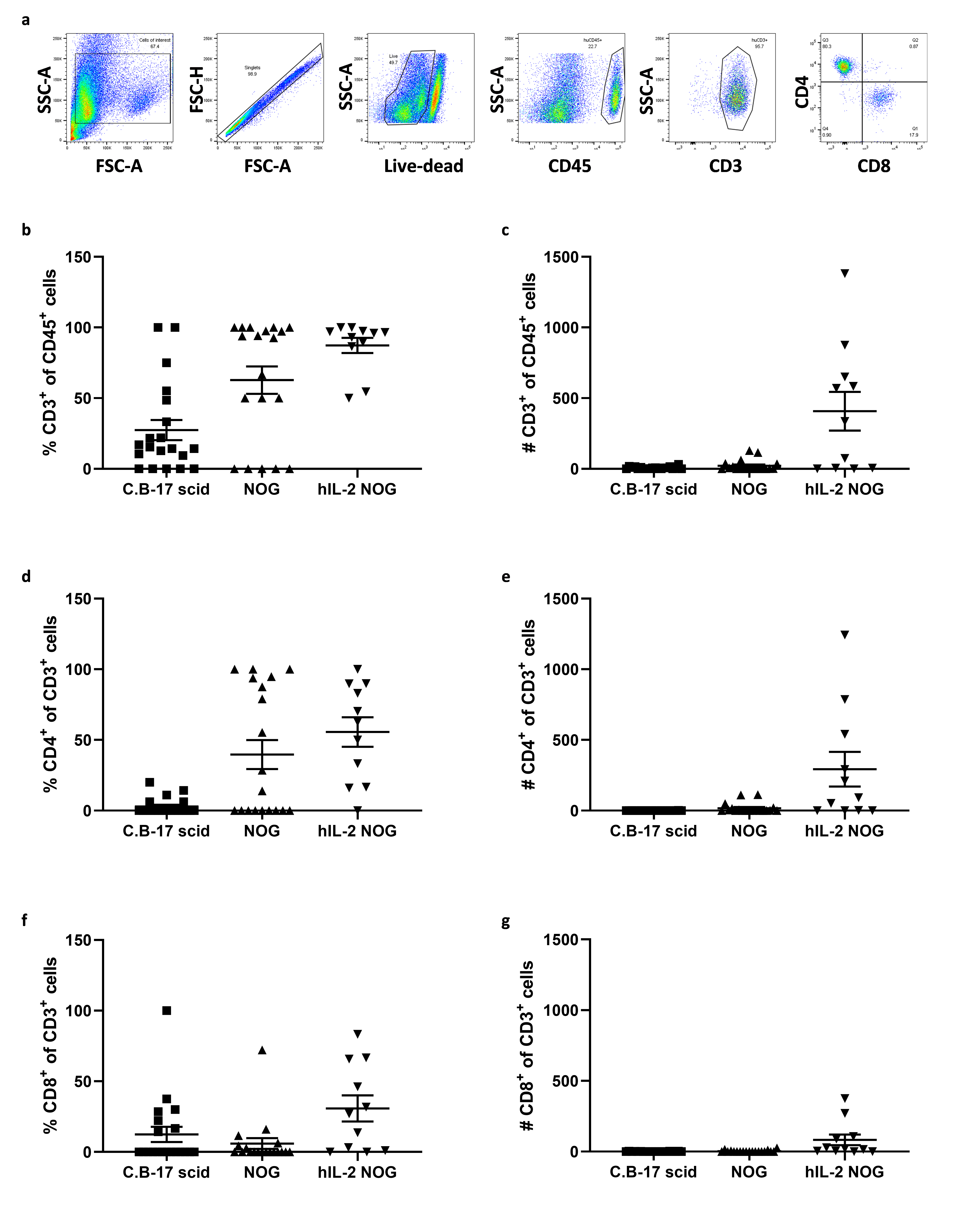

Supplement: S2 Fig — (a) Cells of interest was gated on the forward-side scatter. Of these, singlets were identified and live cells were gated from singlets. Next, human CD45+ cells were gated from live cells and from these CD3+ cells were identified. Lastly, CD4+ and CD8+ cells were gated from CD3+ cells. (b) percent and (c) number of human CD3+ cells of CD45+ cells, (d) percent and (e) number of human CD4+ cells of CD3+ cells and (f) percent and (g) number of human CD8+ cells of CD3+ cells in lymph node cell suspensions. Keratomes from six psoriasis vulgaris patients were included in the study. The number of mice in each group were: C.B-17 scid n = 20; NOG n = 19; hIL2-NOG n = 11. (TIF) [file pone.0278390.s002.tif]

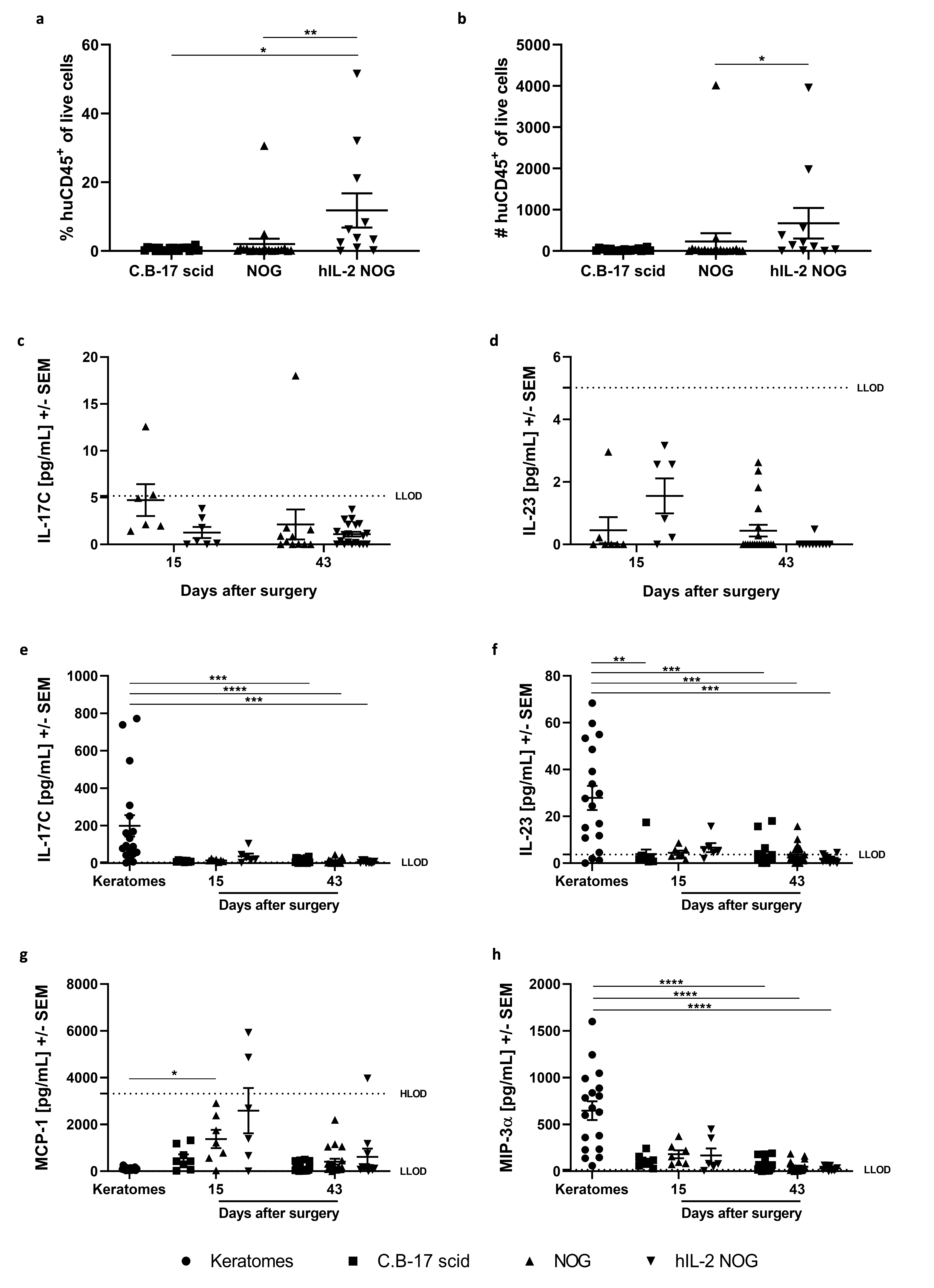

Supplement: S3 Fig — Percent (a) and number (b) of human CD45+ cells of live cells in lysed blood. The gating strategy used was similar to the one used for lymph node cell suspensions. Human IL-17C and IL-23 were analysed in serum (c, d), keratomes and graft lysates (e, f) on day 15 and 43 by the MSD platform. Human MCP-1 (g) and MIP-3α (h) protein levels in keratomes and graft lysates from C.B-17 scid, NOG and hIL-2 NOG mice. Keratomes from six psoriasis vulgaris patients were included. The number of mice in each group was on day 15: C.B-17 scid n = 8; NOG n = 7; hIL2-NOG n = 6 and on day 43: C.B-17 scid n = 20; NOG n = 20; hIL2-NOG n = 11. Lysates were generated from biopsies obtained from keratomes at arrival (three biopsies per keratome, n = 18) and grafts 15 and 43 days after surgery. Serum was isolated from blood obtained on day 15 and 43. LLOD is lower limit of detection and HLOD is higher limit of detection. (TIF) [file pone.0278390.s003.tif]

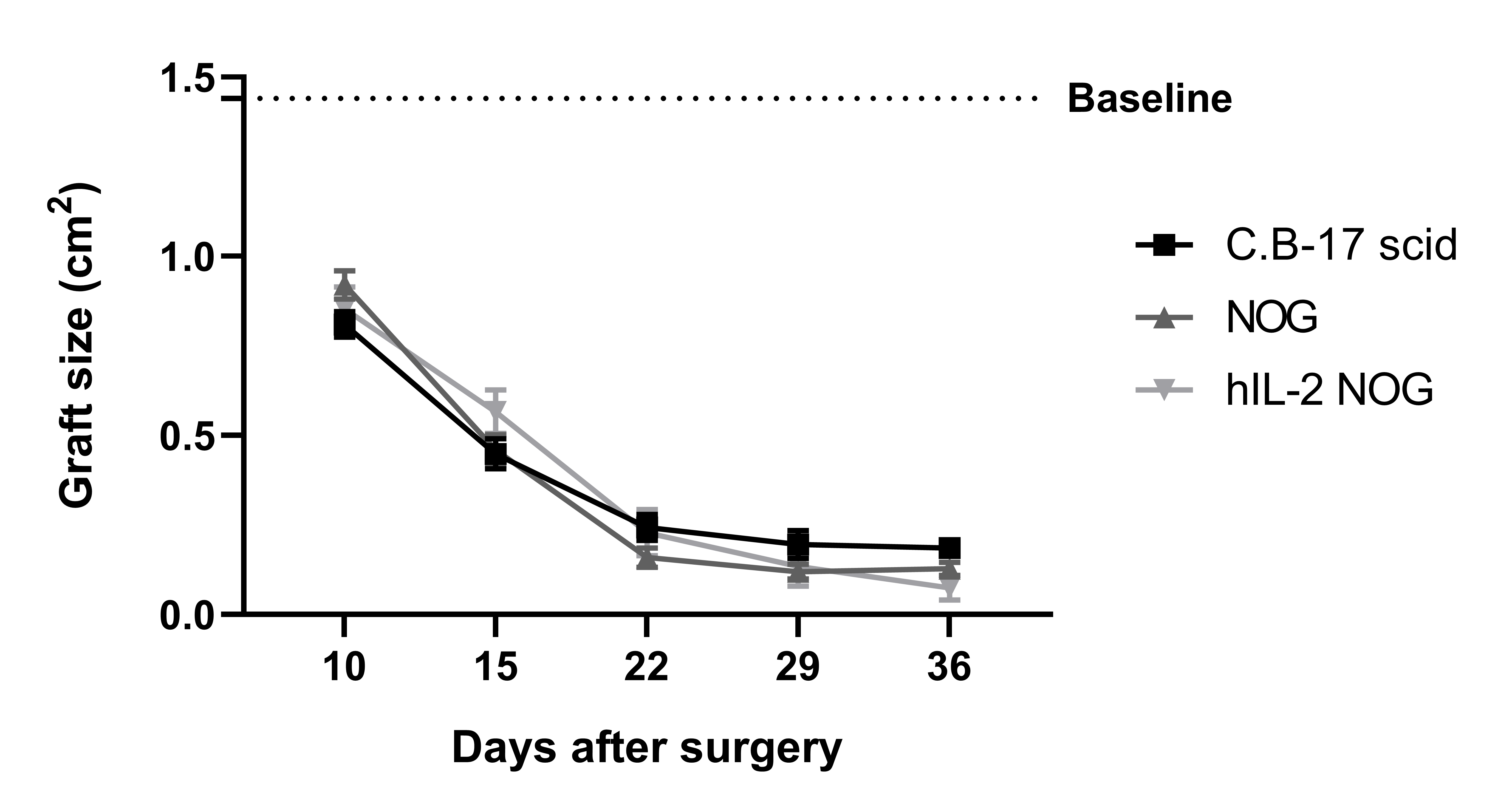

Supplement: S4 Fig — Graft size was evaluated on day 10, 15, 22, 29 and 36 by measuring the length of two sides of the graft. The baseline (dotted line) is the size of the keratome biopsy transplanted onto the mice on the day of the surgery. All mice were included until day 15. As some mice were terminated on day 15 and pre-maturely, fewer mice were included from day 22–36. C.B-17 scid n = 29 (day 10), n = 29 (day 15), n = 20 (day 22), n = 20 (day 29) and n = 20 (day 36). NOG n = 27 (day 10), n = 26 (day 15), n = 20 (day 22), n = 20 (day 29) and n = 20 (day 36). hIL-2 NOG n = 27 (day 10), n = 27 (day 15), n = 15 (day 22), n = 13 (day 29) and n = 11 (day 36). (TIF) [file pone.0278390.s004.tif]

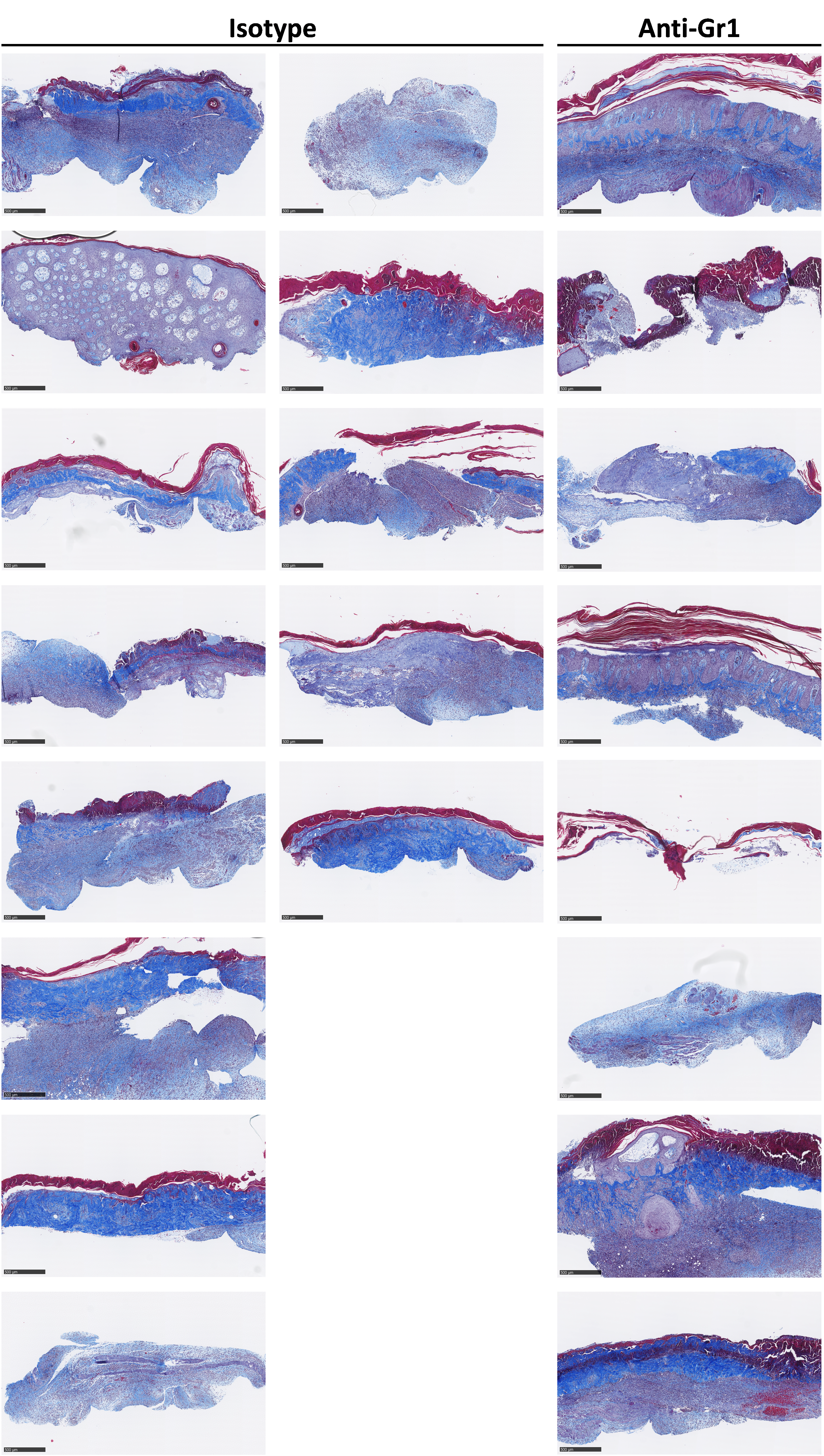

Supplement: S5 Fig — Masson’s Trichrome staining of grafts from hIL-2 NOG mice treated with either rat IgG2b isotype control (n = 13) or anti-mouse GR1 antibody (n = 8). The bar equals to 500 μm and slides are shown in a 5X magnification. (TIF) [file pone.0278390.s005.tif]

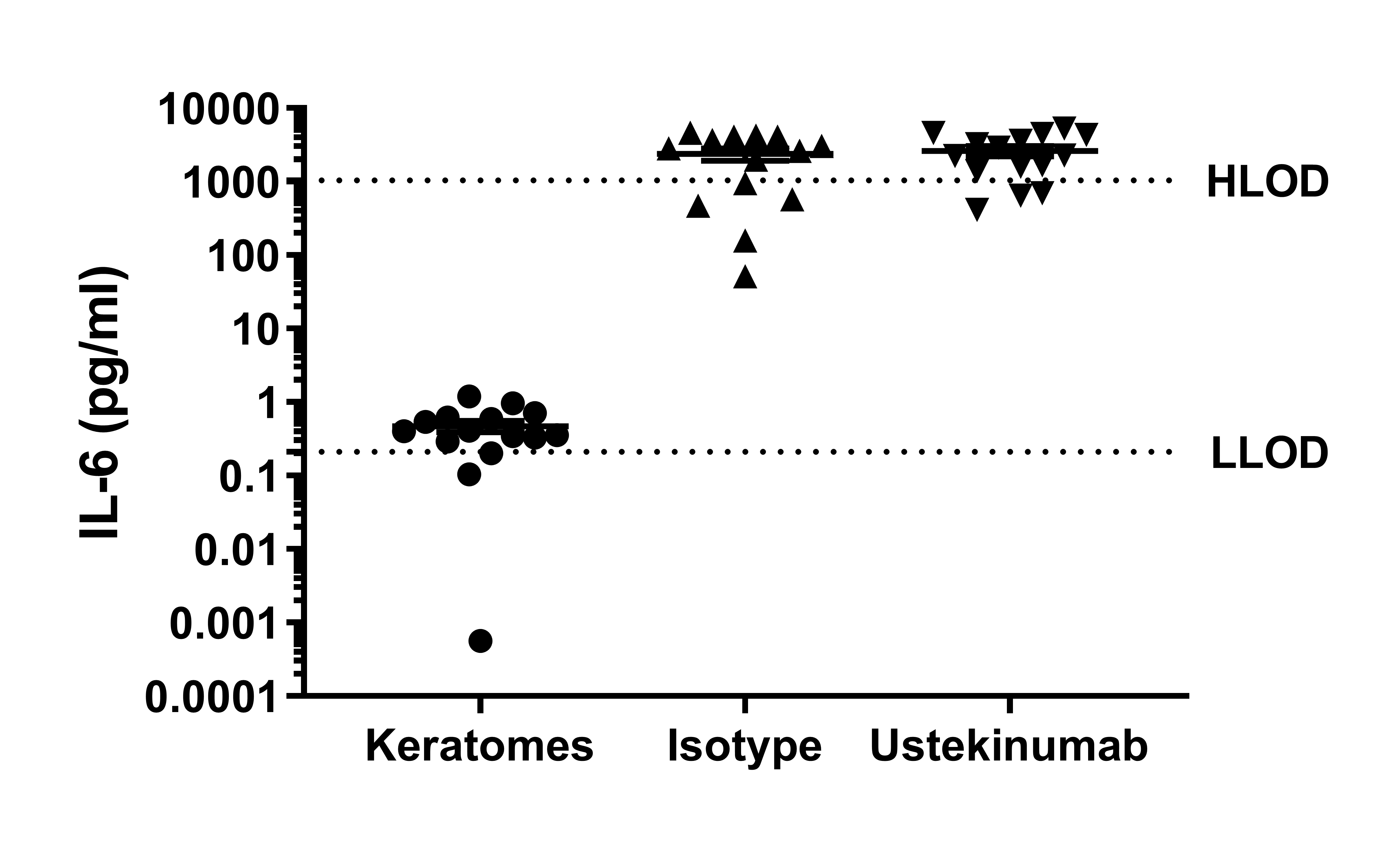

Supplement: S6 Fig — Protein levels of human IL-6 were analysed in keratome (Five psoriasis vulgaris patients were included in the study and three biopsies were obtained per keratome for MSD analyses, n = 15) and graft lysates from hIL-2 NOG mice treated with either ustekinumab or isotype control. The number of mice were n = 14 (isotype) and n = 15 (ustekinumab). LLOD is lower limit of detection and HLOD is higher limit of detection. (TIF) [file pone.0278390.s006.tif]
