# Supplementary material for: Sustaining the T-cell activity in xenografted psoriasis skin
Source: PLoS One. 2023 Jan 17;18(1):e0278390. doi: 10.1371/journal.pone.0278390 (PMC9844869; doi:10.1371/journal.pone.0278390)
Supplement: Attachment — Submitted filename: Rebuttal letter.docx [file pone.0278390.s007.docx]

*Dear Nicholas A. Pullen and reviewers,*

*Thank you for your great and constructive suggestions on how to improve the manuscript. Based on your feedback we have to the best of our abilities revised the manuscript. In the revised version we have tried to address all of the concerns in the best possible way.*

*The changes incorporated in the revised manuscript are:*

- *Additional discussion*
- *Editing of the prose*
- *Journal requirements*

*We hope that you will find the revised manuscript improved and suitable for publication in PLOS ONE.*

*Please do not hesitate to contact me if you have any further questions or comments.*

*Kind regards,*

*Pernille Christensen*

**Journal Requirements:**

When submitting your revision, we need you to address these additional requirements.

1. Please ensure that your manuscript meets PLOS ONE's style requirements, including those for file naming. The PLOS ONE style templates can be found at 
<https://journals.plos.org/plosone/s/file?id=wjVg/PLOSOne_formatting_sample_main_body.pdf> and 
<https://journals.plos.org/plosone/s/file?id=ba62/PLOSOne_formatting_sample_title_authors_affiliations.pdf>

- *We have to the best of our abilities ensured that the manuscript meets PLOS ONE’s style requirements.*

2. To comply with PLOS ONE submissions requirements, in your Methods section, please provide additional information regarding the experiments involving animals and ensure you have included details on (1) methods of sacrifice, (2) methods of anesthesia and/or analgesia, and (3) efforts to alleviate suffering.

- *Thank you for your comment. The required information has now been included in the methods section (line 65-69).*

3. Thank you for stating the following financial disclosure:

"This work was funded by LEO Pharma A/S and the Innovation fund, Denmark (grant number 5189-00097B)."

Please state what role the funders took in the study.  If the funders had no role, please state: ""The funders had no role in study design, data collection and analysis, decision to publish, or preparation of the manuscript."" If this statement is not correct you must amend it as needed.

Please include this amended Role of Funder statement in your cover letter; we will change the online submission form on your behalf.

- *The amended Role of Funder has now been included in the cover letter.*

4. Thank you for stating the following in the Competing Interests section:

"I have read the journal's policy and the authors of this manuscript have the following competing interests: P.K.F.C and M.H.H.-H. are former employees of LEO Pharma A/S. J.K is an employee of LEO Pharma A/S. J.L. is an employee of Bioneer A/S. K.E. is an employee of Amniotics AB. "
  
We note that one or more of the authors are employed by a commercial company: LEO Pharma A/S., Bioneer A/S., and Amniotics AB.

a. Please provide an amended Funding Statement declaring this commercial affiliation, as well as a statement regarding the Role of Funders in your study. If the funding organization did not play a role in the study design, data collection and analysis, decision to publish, or preparation of the manuscript and only provided financial support in the form of authors' salaries and/or research materials, please review your statements relating to the author contributions, and ensure you have specifically and accurately indicated the role(s) that these authors had in your study. You can update author roles in the Author Contributions section of the online submission form.

Please also include the following statement within your amended Funding Statement. 
“The funder provided support in the form of salaries for authors [P.K.F.C, M.H.H.-H. ,J.K, J.L., K.E.], but did not have any additional role in the study design, data collection and analysis, decision to publish, or preparation of the manuscript. The specific roles of these authors are articulated in the ‘author contributions’ section.”

If your commercial affiliation did play a role in your study, please state and explain this role within your updated Funding Statement.

b. Please also provide an updated Competing Interests Statement declaring this commercial affiliation along with any other relevant declarations relating to employment, consultancy, patents, products in development, or marketed products, etc.

Within your Competing Interests Statement, please confirm that this commercial affiliation does not alter your adherence to all PLOS ONE policies on sharing data and materials by including the following statement: ""This does not alter our adherence to PLOS ONE policies on sharing data and materials.” (as detailed online in our guide for authors <http://journals.plos.org/plosone/s/competing-interests>). If this adherence statement is not accurate and there are restrictions on sharing of data and/or materials, please state these. Please note that we cannot proceed with consideration of your article until this information has been declared.

Please include both an updated Funding Statement and Competing Interests Statement in your cover letter. We will change the online submission form on your behalf.

- *The funding statement and competing interest statement has been updated and included in the cover letter.*

5. Please review your reference list to ensure that it is complete and correct. If you have cited papers that have been retracted, please include the rationale for doing so in the manuscript text, or remove these references and replace them with relevant current references. Any changes to the reference list should be mentioned in the rebuttal letter that accompanies your revised manuscript. If you need to cite a retracted article, indicate the article’s retracted status in the References list and also include a citation and full reference for the retraction notice.

- *The reference list has now been corrected and should meet all requirements.*

**Reviewer 1**

*Dear Reviewer 1,*

*Thank you very much for reviewing our manuscript and for finding our work well-conducted and interesting. We have addressed your comments to the best of our abilities. Please find our answers below.*

*Best regards,*

*Pernille Christensen*

Christensen et al. transplanted human psoriasis skin xenografts onto human IL-2 NOG, C.B-17 scid, and NOG mice. The expression of human IL-2 in human IL-2 NOG mice not only sustains T cell activity in the xenografts but also promotes T cells to migrate from the xenografts into the recipient circulation. Therefore, transplantation of human psoriasis skin xenografts induces GVHD in the human IL-2 NOG recipient mice.

The authors also indicate that this model needs further investigations to more accurately model psoriasis.

Comments

The manuscript is well written. The experimental design is elegant, and the experiments are properly executed. The findings are interesting.

Please discuss briefly why T cells in the human psoriasis skin xenografts can induce GVHD in human IL-2 NOG recipient mice. T cells in psoriasis skins may consist of autoimmune, tissue resident, and bystander T cell populations.

- *Thank you for your suggestion. A short discussion of this matter has been included in the revised manuscript (line 362-365).*

In lines 244 and 245, the authors indicate that “the inflammation in the grafts were lower than in serum”. What is serum inflammation? The sentence can be changed into “the inflammation markers in the grafts were lower than in serum”.

- *Thank you for making us aware of this unclarity. The sentence has now been changed according to your suggestion (line 255).*

**Reviewer 2**

*Dear Reviewer 2,*

*Thank you very much for reviewing our manuscript and for your highly relevant comments and suggestions. We have addressed each point to the best of our abilities. Please find our answers below.*

*Best regards,*

*Pernille Christensen*

The authors established a murine xerografted psoriasis model by using human IL-2(hIL-2) NOG mice. Compared with C.B-17 scid and NOG mice, their model showed more T cells in the skin grafts and axillary lymph nodes. Also, higher levels of IL-22, IFN-γ, and TNF-α were observed in the hIL-2 NOG mice model. However, there was lack of efficacy of ustekinumab in terms of inhibition of IL-17A, IL-22, IFN-γ, and TNF-α. Thus, this hIL-1 NOG mrine model of psoriasis needs further investigations to validate its value despite of sustained T-cell activity.

I have some comments and suggestions.

1. Generally speaking, the structure of Results section is not well-organized. Some paragraphs in the Results section are actually figure legends. The description for results in the main text should be different from figure legends. Please separate figure legends (i.e. Line 179, 228, 270, 314, 326) from the main text. Also, some figure panels are not described in the main text. For example, the first paragraph of Results section only describes Figure 1d.

- *We are sorry that you find the results section unstructured. We have to the best of our abilities changed the figure legends and paragraphs to separate the figure legends from the main text (line 183, 239, 282, 559). Furthermore, figure 1 has been changed so the first graph mentioned in the text is Fig 1a.*

2.Some sentences in the manuscript have unclear meaning. The reviewer suggests the authors to sent the manuscript to a native English speaker for English editing.

- *Thank you for your comment. We have changed some of the sentences and upon acceptance of the manuscript we intent to send it to one of PLOS ONEs language editing partners.*

3.Resolution of figures are too low.

- *Thank you for your comment. The resolution of all the figures has now been improved. Furthermore, the figures have been uploaded to and accepted by PACE digital diagnostic tool.*

4.The format of references are not unified. Volume and issue numbers are missing in some references. For example, references 1,2,3,7. In addition, it is unnecessary to indicate the pulisher/associations of these journals. For example, in reference 2, no need to mention the Society for Investigative Dermatology, which publishes the Journal of investigative dermatology.

- *The references have now been adjusted to make them unified.*

5.Figure 1e, the authors compare survival curves of C.B-17 scid, NOG, and hIL-2 NOG mice. However, only two survial curves are shown. Also, the authors didn't indicate which curves stand for which strains.

- *Thank you for noticing this. Two of the survival curves are placed on top of each other which in the graph is marked by both a square and a triangle. This has now been explained in the figure text (line 199-200).*

*The identification of the different strains in the graph has been moved from the left side of the figure to the bottom to make it clearer that this description goes for all the graphs in the figure.*

6.Line 248, why the authors investigate protein levels of MCP-1 and MIP-3α. What is the significance?

- *Thank you for your highly relevant question. The protein levels of MCP-1 and MIP-3α were investigated to evaluate the downstream effect of the human cytokines. This explanation has now been included in the revised manuscript (line 259-260).*

7.Figure 3b, what is the Y-axis? "# of mice"?

- *We are sorry for the confusion. The figure legend has now been changed to “Number of mice”.*
